# Epicardial Adipose Tissue Accumulation and Essential Hypertension in Non-Obese Adults

**DOI:** 10.3390/medicina55080456

**Published:** 2019-08-09

**Authors:** Donatas Austys, Andrej Dobrovolskij, Valerija Jablonskienė, Valerij Dobrovolskij, Nomeda Valevičienė, Rimantas Stukas

**Affiliations:** 1 Department of Public Health, Institute of Health Sciences, Faculty of Medicine, Vilnius University, LT-03101 Vilnius, Lithuania; 2 Department of Radiology, Nuclear Medicine and Medical Physics, Institute of Biomedical Sciences, Faculty of Medicine, Vilnius University, LT-08406 Vilnius, Lithuania; 3 Gemeinschaftsklinikum Mittelrhein gGmbH, Academic Educational Hospital of the Johannes Gutenberg University of Mainz, 56073 Koblenz, Germany; 4 Department of Physiology, Biochemistry, Microbiology and Laboratory Medicine, Institute of Biomedical Sciences, Faculty of Medicine, Vilnius University, LT-03101 Vilnius, Lithuania

**Keywords:** epicardial fat, primary hypertension, grade of hypertension, risk assessment, adults

## Abstract

*Background and Objectives*: Epicardial adipose tissue (EAT) is shown to be an important factor in the development of coronary artery disease, but numerous pathophysiological mechanisms of its action are still only partially understood. There is a lack of studies on its association with different grades of essential hypertension (EH). Therefore, we aimed to evaluate the association between size of EAT depots and the risk of EH taking into account its grade. *Materials and Methods*: Non-obese adult patients with various cardiovascular diseases were investigated: 157 of them had essential hypertension and 101 did not. Hypertensive patients were assigned to three groups according to the grade of hypertension. EAT volume and thickness on ventricular free walls (6 locations) and grooves (5 locations) were measured using cardiac magnetic resonance imaging and compared between groups. A regression model for the prediction of EH was constructed. *Results*: In general, thickness (in all locations) and volume of EAT depots was greater among hypertensive patients than in normotensive (NORM) group. Mean EAT thickness in all 11 locations and EAT volume were lower in NORM than in grade 1 hypertension group; similarly, EAT volume was lower in grade 1 than in grade 2 hypertension group. EAT accumulation did not differ between grade 2 and severe hypertension groups. EAT volume, dyslipidaemia status, body mass index, and age were independent predictors for EH in regression model. *Conclusions*: EAT accumulation is larger among hypertensive than normotensive individuals. Measurement of EAT depots could be beneficial for identification of hypertensive patients and prediction of hypertension severity.

## 1. Introduction

Essential hypertension (EH) is one of the most important modifiable risk factors for coronary heart disease, stroke, congestive heart failure, end-stage renal disease, and peripheral vascular disease. Uncontrolled EH may lead to the detriment of the cardiovascular system, brain, and kidneys [1,2,3]. EH is associated with a variety of non-modifiable and modifiable risk factors [1,4].

Obesity is one of those hypertension risk factors that can be modified. Reduction of adipose tissue depots is one way of alleviating hypertension and lowering the likelihood of subsequent major adverse cardiac events [5]. However, the mechanisms of hypertension development are still not fully understood [6,7]. Furthermore, studies have revealed that not only the amount of adipose tissue but also its distribution is important for the occurrence of cardiovascular diseases, including hypertension. Visceral adipose tissue was found to be more metabolically active and associated with higher risk than superficial subcutaneous adipose tissue [8,9].

Besides abdominal depots of visceral adipose tissue, present studies take into account epicardial adipose tissue (EAT) as a new modifiable cardiometabolic risk factor, which might interact with the heart in a more direct way. EAT is identified as visceral brown adipose tissue located between the myocardium and visceral pericardium and covers more than three quarters of the surface of the heart [10]. Studies have shown that an increase in the size of EAT depots is associated with dysfunction of this tissue, inflammatory processes, cardiac arrhythmias, lipotoxic cardiomyopathy [11], increased calcium scores in the arteries [12,13,14] and coronary artery disease (CAD) [13,14,15,16]. EAT has been shown to be an important factor in the development of CAD, but numerous mechanisms of its action are still only partially understood [17]. Investigation of the association between EAT and CAD risk factors, such as hypertension, might help to describe the development of CAD and other cardiovascular diseases in more detail. On the other hand, determination of real hypertensive patients remains a clinical problem, and finding the imaging technique to distinguish hypertensive patients from those with normal blood pressure would be beneficial [18]. Few studies were performed in order to investigate the association between hypertension and the size of EAT depots, but different methods and diverging results do not provide clear evidence for such an association [13,14,18,19,20,21,22,23,24,25,26,27,28]. In addition to this, some studies indicate the association between the size of EAT depots and severity of hypertension [21,25,29]. However, we found no studies regarding the possibility to use the size of EAT depots for the prediction of different grades of hypertension. In addition, no English-written articles about EAT depots of residents of Lithuania have been published before.

The objective of this study was to evaluate the association between the size of epicardial adipose tissue depots and the risk of essential hypertension with respect to its grade.

## 2. Materials and Methods

### 2.1. Population of the Study

The study involved adults between the ages of 30 and 75. According to their health records, they were assigned to normotensive (NORM) and hypertensive groups. Diagnoses of essential hypertension and its grade were made by health care professionals according to Guidelines for the Management of Arterial Hypertension [4]. People with up to 139 mmHg systolic and up to 89 mmHg diastolic office blood pressure and no indications for hypertension in their health records were assigned to the NORM group. It consisted of 101 patients. The second group consisted of 157 patients who had a diagnosis of essential hypertension, but had no secondary hypertension, diabetes mellitus, thyroid, or kidney disease. Hypertensive patients were assigned to three groups according to the grades of hypertension: grade 1 hypertension (EH1), grade 2 hypertension (EH2) and grade 3 hypertension (EH3) [4].

To exclude any confounding effect on the study results, all obese (body mass index greater than 29.9 kg/m^2^) individuals were excluded from this study. 

The study was conducted according to the Vilnius Regional Biomedical Research Ethics Committee permission (No: 158200-13-576-178, supplement No: 158200-576-PP1-14). All participants of this study gave their informed consent prior to their inclusion in the study.

### 2.2. Assessment of the Lifestyle

To assess lifestyle factors that might be associated with EH, a questionnaire was used. Hypertension risk factors such as low physical activity, frequent emotional stress, hard smoking, high salt intake, and a frequent consumption of butter and animal fats were investigated. Physical activity assessment included physical activity at work and leisure time physical activity. Low physical activity group consisted of individuals with low physical activity at work and during leisure time. Sedentary work as well as work including only walking or standing was assigned to low physical activity. Similarly, leisure time physical activity including only sitting, standing or walking was defined as low. Frequent emotional stress group was formed of individuals who subjectively indicated at least one of the following: frequent feeling of tension and anxiety, frequent feeling of suppression and slowness, frequent torment of troubled thoughts and concerns, rare ability to sit calmly and relax. At least 10 pack-years current or ex-smokers were identified as hard smokers. Excessive salt consumption group was formed of individuals who indicated additional salting of prepared meals. Participants who indicated animal fats or butter as the most frequently consumed dietary fats were assigned to another risk group.

### 2.3. Assessment of the Size of Epicardial Adipose Tissue Depots

**Figure 1:**
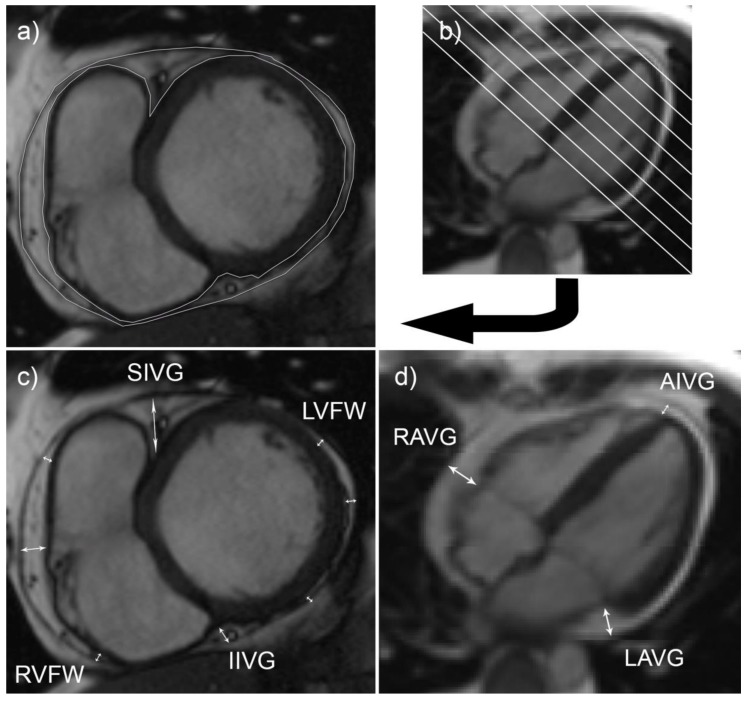
EAT measurements on cardiac magnetic resonance imaging. True fast imaging with steady-state precession (TrueFISP) images of horizontal long-axis (**b**), (**d**) and short-axis (**a**), (**c**) views in end-diastole were used. (**a**) and (**b**) images show measurement of EAT volume: an outlined EAT area (**a**) in one of the slices (**b**). (**c**) and (**d**) images show measurement of EAT thickness on the right ventricular free wall (RVFW), left ventricular free wall (LVFW), in the superior interventricular groove (SIVG), inferior interventricular groove (IIVG), anterior interventricular groove (AIVG), right atrioventricular groove (RAVG), left atrioventricular groove (LAVG).

The size of EAT depots was measured using cardiac magnetic resonance tomography images. Cardiac magnetic resonance imaging was performed for each participant. Volume and thickness of EAT was measured (Figure 1).

Volume of EAT was calculated by using modified Simpson rule [30]. End-diastolic short axis images were used for the EAT measurements. Depots of EAT on left and right ventricles were outlined by hand on each slice. Volume of epicardial adipose tissue was calculated by summing up all area measurements and by multiplying the sum by the distance between slices (8 mm).

Thickness of EAT depots was measured in 11 locations. End-diastolic long-axis (4-chamber) images were used for measurements of EAT thickness in right and left atrioventricular grooves and the anterior interventricular groove. End-diastolic short-axis images were used to measure thickness of EAT depots on the free walls of left and right ventricles as well as in superior and inferior interventricular grooves.

### 2.4. Assessment of other Variables

On admission to our hospital, blood pressure (BP) of every patient was measured. As recommended [4], measurements at the upper arm were performed in a quiet room, with the patient in the seated position, back and arm supported, after 5 min of rest. In the event of a significant (>10 mmHg) difference between arms, the arm with the higher BP values was used.

Also, use of statins and antihypertensive drugs within the period of one year was recorded. Drugs were assigned to groups as follows: beta-blockers, diuretics, angiotensin-receptor blockers, calcium channel blockers, ACE inhibitors, and other antihypertensives.

In addition, within one year to magnetic resonance tomography measurements of total cholesterol, low-density lipoprotein cholesterol (LDL-c), high-density lipoprotein cholesterol (HDL-c), and triglycerides (TG) were made.

### 2.5. Statistical Analysis of Data

In statistical analysis, mean values of EAT thickness were used as follows: mean of all thicknesses (measurements in 11 locations), mean thickness of groove measurements (5 locations), mean thickness of atrioventricular groove measurements (2 locations), mean thickness of interventricular groove measurements (3 locations), mean thickness of measurements on ventricular free walls (6 locations), mean thickness of measurements on the free wall of the right ventricle (3 locations), and mean thickness of measurements on the free wall of the left ventricle (3 locations).

Normality of the variables’ distribution was tested using the Shapiro–Wilk test. A *T*-test was used for the comparison of means with normal distribution of variables. The Mann–Whitney *U* test was applied for the comparison of variables that did not comply with the assumptions of normality. Frequencies between the groups were compared using the χ^2^ criterion. Receiver operating characteristic (ROC) curve analysis was performed in order to identify optimal cut-values for continuous variables. A binary logistic regression model was constructed for the prediction of EAT volume. Only statistically significant variables were used for the final model. Odds ratios and confidence intervals were calculated for each variable of the model. Hosmer–Lemeshow goodness of fit test was applied. Next, differences of EAT volume between grades of hypertension were assessed by analysis of covariance (ANCOVA) with body mass index (BMI), pack-years and sex as covariates. Because of the small sample size, patients with grade 3 hypertension were excluded from ANCOVA analysis. Significance level α = 0.05 was chosen for statistical analysis. Measures of central tendency are presented as follows: mean ± standard deviation, for variables with normal distribution, or median (first quartile–third quartile) for variables with other distribution. 

## 3. Results

Despite our efforts to create groups of patients as similar as possible in terms of essential hypertension risk factors, they did have some differences. Hypertensive patients were older and had slightly higher BMI than the NORM group; a larger part of hypertensive group were also male and hard smokers. On admission, NORM group had lower BP and TG levels, and less of them used antihypertensives. On the other hand, there was no significant difference in emotional stress experiences, physical activity, and consumption of salt and dietary fats (Table 1). On admission to our hospital, systolic BP in EH1, EH2, and EH3 groups respectively was 130.6 ± 14.6 mmHg, 139.5 (127.8–150.0) mmHg, and 158.4 ± 18.5 mmHg. Diastolic BP in these groups was 81.1 ± 13.7 mmHg, 80.0 (80.0–90.0) mmHg, and 93.4 ± 10.8 mmHg.

Overall, median EAT volume and thickness were 125.5 cm^3^ (100.3–153.4 cm^3^) and 5.4 mm (4.5–6.2 mm) respectively. Analysis showed that the size of EAT depots (all measurements) were statistically significantly higher among hypertensive patients than in NORM group (*p*-value < 0.001). Respectively, median EAT volume was 137.5 cm^3^ (113.1–159.5 cm^3^) and 108 cm^3^ (89–137 cm^3^), median EAT thickness was 5.7 mm (4.8–6.4 mm) and 4.9 mm (4.3–5.8 mm). Differences in size of EAT depots between NORM and EH1 groups were observed only when comparing volume, average values of all thicknesses and average values of all thicknesses in grooves. Differences between EH1 and EH2 were also observed when comparing volume, as well as average values of all thickness measurements. In addition to this, the size of EAT depots between EH1 and EH2 groups differed when comparing the average values of EAT thickness on the free ventricular walls (similarly only on the right ventricular wall). There was no difference in size of EAT depots between EH2 and EH3 groups. Detailed analysis of the size of EAT depots in different groups of patients is presented in Table 2.

The ROC analysis showed that volume, among all EAT measurements, provides the most accurate results for identification of hypertensive patients (area under the curve was 0.687). EAT volume is also applicable for identification of EH1 patients in a mixed EH1 and NORM group. The mean EAT thickness in all measurement locations accounted for 0.669 area under the curve for identification of hypertensive patients in the whole sample.

According to the ROC analysis, optimal EAT volume cut-point (Youden’s Index) to indicate EH patients was 111.6 cm^3^. Optimal EAT thickness cut-points for average of all thicknesses, average of measurements in grooves, on both ventricular free walls, and on the right ventricular free wall were 4.8 mm, 6.9 mm, 2.8 mm, and 4.2 mm, respectively. We used these values in order to conduct logistic regression analysis and to construct a model which showed that EAT volume is an independent determinant of EH and along with age, BMI, and dyslipidaemia status, can be used to identify hypertensive patients. Furthermore, logistic regression analysis showed no significance for any EAT thickness measurements, whether analysing with or without the EAT volume variable. The model of the independent determinants of EH prediction is provided in Table 3. These determinants, except for the BMI, remained significant after adding antihypertensive drugs to the model.

After adjusting for sex, BMI, hard smoking, statins, and antihypertensives in ANCOVA, it was found that grade of hypertension was a significant determinant for EAT volume. According to this analysis, EAT volume differed significantly between EH2 and EH1, also between EH2 and NORM groups (respectively, *p* = 0.039 and *p* = 0.003). No difference in EAT volume was found between NORM and EH1 groups. Age and dislipidaemia status were not significant covariates for EAT volume.

## 4. Discussion

Results of this study show that the size of EAT depots tend to be higher among the patients with EH compared to normotensive patients. Also, this study shows that EAT depots tend to increase up to the second grade of hypertension while patients with severe hypertension tend to have similar EAT depots to that of patients with grade 2 hypertension. Furthermore, logistic regression analysis shows that EAT volume is an independent determinant of EH and can be used for its risk prediction in conjunction with such variables as age, body mass index and dyslipidaemia status, improving the prediction accuracy. According to ANCOVA, after adjustment for sex, BMI and hard smoking, the grade of hypertension remains significant determinant for EAT volume.

Some previous studies conducted by other authors confirm the increase in size of EAT depots among the patients with EH [18,19,31,32,33,34,35]. The difference between our study and the majority of previous studies is that along with EAT thickness measurements we have also measured EAT volume. In addition to this, cardiac magnetic resonance tomography method was used for our measurements which is considered to be the gold standard for EAT measurements. According to other authors, volumetric measurements of EAT are more accurate than EAT thickness measurements but there exist technological and time limitations for measurement of EAT volume [36]. Our study confirms the advantage of volumetric EAT measurements. Despite our efforts to find a location (or group of locations) for EAT thickness measurements that could be used instead of EAT volumetric measurements for EH risk assessment, logistic regression analysis showed that EAT thickness (in those 11 locations we defined) realistically cannot be used for EH risk prediction. Accordingly, it may be beneficial to improve methodologies to increase accuracy of EAT thickness measurements as they can be performed using cheaper and more accessible techniques.

Contrary to our findings, some studies have shown no association between EAT and hypertension [21,23,24,27,28]. Wang et al. found no association between EAT volume and blood pressure among 49 patients with type 2 diabetes mellitus and 78 nondiabetic controls [28]. Another group of scientists did not find a significant difference in prevalence of hypertension among 93 patients with chronic kidney failure grouped by a median EAT volume [24]. Similarly, no significant difference in the prevalence of hypertension was found among patients with CAD stratified by median EAT thickness [27] or EAT volume quartiles [23]. The authors from the Republic of Korea stated that the average EAT thickness on the right ventricular free wall differed significantly between hypertensive and normotensive females, whereas in males, no significant difference was observed. Furthermore, logistic regression analysis performed by these researchers showed that EAT is an independent determinant of nocturnal non-dipping blood pressure pattern for females, while for males it was not significant [21]. Our analysis showed that EAT thickness might not be accurate enough to predict hypertension. Moreover, ROC analysis showed that the optimal cut-point for classification of normotensive and hypertensive patients according to EAT volume might not be equal to the median EAT volume. Therefore, median values of EAT depot size should be avoided when demonstrating the absence of association between EAT and hypertension as we found in some other studies [24,27].

Along with EH occurrence disparities, diverging results regarding the correlation between the size of EAT depots and blood pressure are presented in the literature [19,26]. We have found no studies where EAT depots would be assessed with respect to the grade of hypertension that describes the state of a patient more accurately than momentary blood pressure measurements.

In the literature we have found two theories of the association between EAT and EH. One of these theories states that EAT might be the cause of hypertension. Due to the oxygen deficiency, which occurs when EAT depots become larger, epicardial fat is invaded by increased numbers of macrophages and T lymphocytes, resulting in a shift of its metabolic profile. Increase in EAT depots results in increased secretion of numerous proinflammatory cytokines and vasoactive peptides, including interleukin-6, TNF-α, MCP-1, and angiotensin II [36,37,38]. These substances can, independently of each other, activate renin-angiotensin system and determine the increase in arterial blood pressure and hypertrophy of the left ventricle [37]. In addition to this, an increase in plasma free fatty acid levels may stimulate cardiac autonomic nervous system and subsequently increase arterial blood pressure [39]. Furthermore, EAT might be responsible for hypoadiponectinemia, which is also associated with subsequent increase in blood pressure [40,41]. Erdogan et al. also hypothesised that inflammatory mediators (secreted by EAT), and disruption of local collagen metabolism (induced by myocardial inflammation), might cause myocardial hypertrophy and subsequent hypertension. According to the researchers, these mechanisms might increase left ventricular mass independently of blood pressure values [26].

Another of the theories of the association between EAT and hypertension states that depots of EAT increase because of the adaptation to higher myocardial energy demands. Researchers hypothesize that hypertension might determine the accumulation of EAT. Long-term effect of high blood pressure causes myocyte cellular hyperplasia and capillary proliferation, which subsequently increases thickness of the ventricular wall. This results in increased energy demands for contraction and might stimulate EAT accumulation to provide more free fatty acid to the myocardium [20,25]. Studies show that EAT mass increases along with myocardial mass [42], although myocardial thickness of the left ventricle plays the key role. Our results, similar to the results of other researchers, show that EAT thickness on the right ventricle is higher than on the left ventricle. This is opposite to myocardial thickness, as well as energy demands of the ventricles [42]. As both ventricles are contained in one pericardial sack, it seems to be true that increased energy demands of one ventricle might determine the increase in EAT depots on another with lower energetic needs. This might be related to the differences in size of EAT depots between hypertension grade groups we observed in our study. Furthermore, density of EAT was found to be lower among individuals with hypertension [32].

There were several limitations in our study. Although the sample of our study seems to be sufficiently large compared to other studies on this topic, it was difficult to determine any causal relationship, because predictive factors and outcome variables observed simultaneously without follow-up data. Also, the design of our study did not allow us to assess the significance of hypertension duration to changes in EAT depots; longitudinal studies in the future should address this option.

## 5. Conclusions

Accumulation of EAT among hypertensive patients is larger than among normotensive individuals. Measurement of EAT depots might be beneficial for identification of hypertensive patients and prediction of hypertension severity.

## Figures and Tables

**Table 1:** Characteristics of the study sample and comparison of EH and NORM groups.

Variables	Total (*n* = 258)	EH Group (*n* = 157)	NORM Group (*n* = 101)	*p*-Value
Age median and IQR, years	52 (42–61)	56 (49–65)	43 (36–54)	<0.001
Males ^1^, %	62.0	66.9	54.5	0.045
BMI median and IQR, kg/m^2^	25.0 (22.8–27.5)	26.0 (23.6–27.8)	23.9 (22.0–28.5)	<0.001
Systolic BP median and IQR, mmHg	130.0 (120.0–140.0)	139.0 (128.5–150.0)	120.0 (110.0–131.0)	<0.001
Diastolic BP median and IQR, mmHg	80.0 (71.0–90.0)	80.0 (78.5–90)	79.0 (70.0–80.0)	<0.001
Total cholesterol median and IQR, mmol/L	5.1 (4.2–6.1)	5.1 (4.2–6.1)	5.0 (4.4–6.5)	0.952
LDL-c median and IQR, mmol/L	3.3 (2.6–4.2)	3.3 (2.6–4.1)	3.3 (2.6–4.3)	0.698
HDL-c mean and IQR, mmol/L	1.2 ± 0.4	1.2 ± 0.4	1.2 ± 0.4	0.539
TG median and IQR, mmol/L	1.2 (0.9–1.8)	1.3 (0.9–1.8)	1.0 (0.7–1.3)	0.041
Use of statins, %	35.8	49.7	14.0	<0.001
Use of beta-blockers, %	61.1	70.7	46.0	<0.001
Use of diuretics, %	35.8	44.6	22.0	<0.001
Use of angiotensin-receptor blockers, %	6.6	10.8	0.0	0.001
Use of calcium channel blockers, %	21.0	30.6	6.0	<0.001
Use of ACE inhibitors, %	40.1	59.9	9.0	<0.001
Use of other antihypertensives, %	5.4	8.9	0.0	0.002
Overweight ^1^, %	49.2	59.9	32.7	<0.001
Individuals with dyslipidaemia ^1^, %	47.3	64.3	20.8	<0.001
Low physical activity, %	44.1	41.4	48.5	0.266
Frequent emotional stress ^1^, %	52.3	51.6	48.4	0.772
Excessive salt consumption ^1^, %	26.7	28.7	23.8	0.385
Frequent consumption of butter and animal fats ^1^, %	20.9	19.7	22.8	0.577
Hard smoking ^1^, %	31.8	37.6	22.8	0.013

^1^ Groups with higher risk of cardiovascular diseases.

**Table 2:** Size of EAT depots in NORM, EH1, EH2, and EH3 groups.

Variables	NORM (*n* = 101)	EH1 (*n* = 49)	EH2 (*n* = 88)	EH3 (*n* = 20)	Difference between Groups
EAT volume, cm^3^	108 (89–137)	126.1 ± 29.6	142.4 ± 35.2	142.3 ± 41	a,b,*
Mean EAT thickness (all measurements), mm	4.9 (4.3–5.8)	5.4 ± 1	5.9 ± 1.2	5.8 ± 1.2	a,b,*
Mean EAT thickness in all grooves, mm	6.8 (6–8.7)	7.6 ± 1.5	8.1 ± 1.7	8.2 ± 1.9	a,*
Mean EAT thickness in atrioventricular grooves, mm	7.5 (6–8.5)	8.1 ± 1.8	8.6 ± 2.1	8.8 ± 2.2	*
Mean EAT thickness in interventricular grooves, mm	6.7 (5.7–8.2)	7.2 ± 1.5	7.3 (6.3–9.3)	7.9 ± 1.9	*
Mean EAT thickness on free ventricular walls, mm	2.7 (2.2–3.5)	3 ± 0.9	3.3 (2.8–4.2)	3.4 ± 1	b,*
Mean EAT thickness on the right free ventricular wall, mm	3.7 (3–4.7)	4 ± 1	4.7 (4–5.7)	3.7 (3.4–5.3)	b,*
Mean EAT thickness on the left free ventricular wall, mm	1.3 (1–2.3)	1.7 (1–2.7)	2.2 (1–3.3)	2.7 (1.3–3.7)	*

Results are presented in the following format: mean ± standard deviation for normal distribution, and median (interquartile range) for others. Statistically significant (*p*-value < 0.05) difference between NORM and EH1 group is marked (a), between EH1 and EH2 group is marked (b), between normotensive (NORM group) and all hypertensive patients is marked (*).

**Table 3:** Binary logistic regression analysis to identify the independent determinants of essential hypertension.

Risk Factor	Odds Ratio (95% CI)	*p*-Value
EAT volume > 111.6 cm^3^	1.955 (1.011–3.780)	0.046
Having dyslipidaemia	3.703 (1.940–7.068)	<0.001
BMI > 25.4 kg/m^2^	1.950 (1.006–3.781)	0.048
Age > 47.5 years	4.427 (2.389–8.205)	<0.001

Negelkerke *R* Square 0.398, Cox & Snell R Square 0.294, Hosmer & Lemeshow Test *p* = 0.517, overall correctly predicted percentage 75.4 (with the cut value 0.5). Odds ratios were adjusted for EAT volume, dyslipidaemia status, BMI, age, gender, and hard smoking variables.
